# Social Determinants of Health and Medication Adherence in Older Adults with Prevalent Chronic Conditions in the United States: An Analysis of the National Health and Nutrition Examination Survey (NHANES) 2009–2018

**DOI:** 10.3390/pharmacy13010020

**Published:** 2025-02-07

**Authors:** Omolola A. Adeoye-Olatunde, Tessa J. Hastings, Michelle L. Blakely, LaKeisha Boyd, Azeez B. Aina, Fatimah Sherbeny

**Affiliations:** 1 Department of Pharmacy Practice, College of Pharmacy, Purdue University, West Lafayette, IN 47907, USA; aaina@purdue.edu; 2 Department of Clinical Pharmacy and Outcomes Sciences, University of South Carolina College of Pharmacy, Columbia, SC 29208, USA; hastint@mailbox.sc.edu; 3 Department of Pharmaceutical Sciences, University of Wyoming School of Pharmacy, Laramie, WY 82071, USA; michelle.blakely@uwyo.edu; 4 Department of Biostatistics and Health Data Science, Indiana University School of Medicine and Richard M. Fairbanks School of Public Health, Indianapolis, IN 46202, USA; ljboyd@iu.edu; 5 Institute of Public Health, College of Pharmacy and Pharmaceutical Sciences, Florida A&M University, Tallahassee, FL 32307, USA; fatimah.sherbeny@famu.edu

**Keywords:** medication adherence, social factors, older adults, high blood pressure, high cholesterol, diabetes

## Abstract

Background: The older adult population is rapidly expanding in the United States (US), with a high prevalence of high blood pressure, high cholesterol, and diabetes. Medication nonadherence is prevalent in this population, with less evidence on the influence of social determinants of health (SDoH). Thus, the objective of this study was to identify and prioritize SDoH associated with medication adherence among US older adults with these comorbidities. Method: Using the World Health Organization Commission on Social Determinants of Health and Pharmacy Quality Alliance Medication Access Conceptual Frameworks, publicly available National Health and Nutrition Examination Survey datasets (2009–2018) were cross-sectionally analyzed among respondents aged 65 and older who were diagnosed with study diseases. Data analyses included descriptive statistics, and logistic regression using an alpha level of 0.05. Result: Analyses included 5513 respondents’ data. Bivariate analysis revealed significant differences in medication adherence based on several structural (e.g., ethnicity) and intermediary (e.g., disability status) determinants of health. Multivariable analysis revealed significant differences in medication adherence for alcohol consumption (*p* = 0.034) and usual healthcare place (*p* = 0.001). Conclusions: The study findings underscore pertinent implications for public health and policy, with specific SDoH being the most likely to affect medication adherence in common chronic conditions among older adults in the US.

## 1. Introduction

Improvements in life expectancy have culminated in a population drift toward older adults that is marked by a great prevalence of chronic diseases, often managed by one or more prescription medications [1,2,3,4]. Hypertension, high cholesterol, and diabetes are the prevailing chronic diseases in this population, with 42% of individuals taking five or more prescription medications and a nonadherence rate of 20–60% [5,6,7,8].

Disparities in the prevalence of these diseases were reflected among racial minority groups, with significant contributions from medication nonadherence and Social Determinants of Health (SDoH) [9,10,11,12]. SDoH are “the environmental conditions where people are born, live, learn, work, play, worship, and age that affect a wide range of health, functioning, and quality-of-life outcomes and risks [13]”. However, there have been shortfalls in national concerted efforts to manage SDoH, health disparities, and medication nonadherence concurrently or consistently [13,14].

The Centers for Medicare and Medicaid Services administer adherence-related quality measures for Medicare Part D (prescription medication) insurance policy plans, grading plans on the percent of beneficiaries adherent to hypertension, high cholesterol, and diabetes medications [15]. These plans incentivize pharmacies to drive medication adherence quality measures positively via rebate incentives and by including them in preferred pharmacy networks, ensuring consistent access for patients [16].

Nevertheless, community pharmacists’ inability to address SDoH negatively influences adherence-related quality measures among older adult Medicare beneficiaries [17]. Also, the extant literature has not fully delineated the association between SDoH domains and adherence to concurrent medications for hypertension, high cholesterol, and/or diabetes [18]. The studies identified in this review were narrow in scope, focusing on just one specific disease and a less diverse population. Also, a study on SDoH and adherence to antihypertensive medications has highlighted that most SDoH analyses do not prioritize factors such as health behaviors and social resources like housing and food insecurity [19]. Thus, the primary objective of this study was to identify and prioritize SDoH associated with medication adherence among older adults with hypertension, high cholesterol, and/or diabetes in the US. The hypothesis was that structural and intermediate determinants of health were associated with medication adherence. The secondary objective was to estimate self-reported medication adherence while highlighting implications for pharmacy practice and underserved populations.

## 2. Materials and Methods

### 2.1. Study Design

This cross-sectional study examined a nationally representative sample of secondary data obtained from the National Health and Nutrition Examination Survey (NHANES) database, which included secondary datasets designed to examine the health and nutritional status of a representative sample of US adults and children [20]. Five biannual data years (2009–2018) were downloaded from the NHANES database Ethical approval was not required for the study and was reported using Strengthening the Reporting of Observational Studies in Epidemiology (STROBE) statement guidelines [21].

### 2.2. Study Population

The study population for analysis included all respondents from the 2009–2018 NHANES datasets aged 65 and older whose doctors told them to take at least one prescription for hypertension or cholesterol and/or were told they had diabetes. Post hoc power analysis produced a power greater than 99% with an alpha value of 0.05 when the difference in proportions between the groups was 4% or larger.

### 2.3. Conceptual Framework

**Figure 1:**
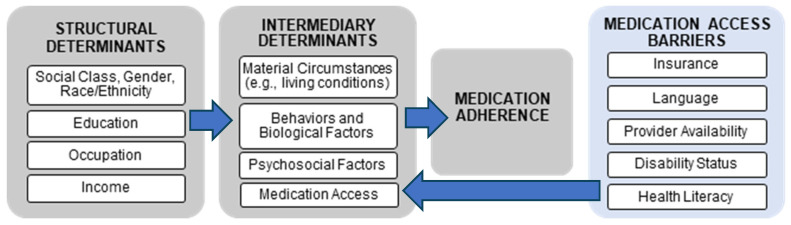
Adeoye-Olatunde et al.’s [23] integrated conceptual framework on social determinants of health and medication adherence. Legend: 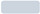 Blue components: Pharmacy Quality Alliance Framework (PQA) 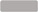 Grey components: World Health Organization Commission on Social Determinants of Health.

Two complementary conceptual frameworks—(1) World Health Organization (WHO) Commission on Social Determinants of Health (CSDH) and (2) Pharmacy Quality Alliance (PQA) Medication Access—were integrated (Figure 1) to inform and categorize SDoH covariates in the NHANES dataset and minimize selection bias [11,22].

Two CSDH elements, structural and intermediate determinants of health, were used for NHANES’ individual-level measurement. The framework defines structural determinants as “social determinants of health inequities”; these inequities function through intermediary determinants—material, psychosocial, behavioral, biological factors, and healthcare access—that mediate the effects of structural determinants on health inequities and directly impact health outcomes. Therefore, structural and intermediary determinants were operationalized as SDoH [22]. Healthcare access and health outcome were redefined as medication access and medication adherence, respectively. Finally, the study added barriers to medication access from the PQA Medication Access framework, which were unaddressed in the CSDH framework [11,22].

### 2.4. Data Variables, Sources, Management and Statistical Methods

Applicable NHANES datasets were combined by study identification number using SAS version 9.4 (SAS Institute, Inc., Cary, NC, USA). Those variables needed for analysis (as defined by the conceptual framework) were retained, while all other variables were eliminated from the combined dataset.

All NHANES datasets used, interpretations, computations, and mapping of study variables to conceptual framework determinants are published in the data dictionary, which is available online at https://doi.org/10.6084/m9.figshare.21947018 (accessed on 2 February 2025). The mapping of study variables to conceptual framework determinants is also depicted in the bivariate results table. Notably, the emergency room was included as a usual place for healthcare to align with the predefined response options. While emergency rooms are typically used for emergent care, this categorization reflects the respondent’s most frequent source of care, consistent with the survey’s intent to capture healthcare utilization patterns. This reflects the lived realities of individuals who may rely on emergency rooms for routine care commonly due to barriers to accessing primary care. Alcohol consumption was categorized as an intermediary SDoH under behavioral and biological factors per the WHO framework. Similarly, age is considered a biological factor that is an intermediary determinant of health.

Descriptive statistics were used to characterize the study population. The study’s outcome variable, medication adherence, was dichotomized into “Adherent” and “Not Adherent”. Respondents were considered adherent if they responded that they were currently taking all prescribed medications for each studied disease state they had (i.e., hypertension, high cholesterol, diabetes) [18,24].

Bivariate analyses utilized logistic regression for continuous predictors and Rao-Scott Chi-Square tests for categorical predictors. Multivariable analysis for medication adherence utilized logistic regression. Predictors with *p*-values less than 0.20 in the bivariate analyses were considered predictors in the multivariable analysis [25]. This *p*-value is often used to include variables that have moderate associations with the outcome variable [25]. Multicollinearity effects were reduced by removing OR ≥ 2.477 corresponding to a Cohen’s d of 0.50. A 5% significance level was used for all tests.

## 3. Results

A total of 5513 respondents met the study’s inclusion criteria. The majority of respondents were 75 years of age or older (46.2%), identified as female (51.7%), Non-Hispanic White (50.6%), and married (54.7%). Hypertension was most prevalent (78.7%), followed by high cholesterol (65.6%) and diabetes (32.8%). Most respondents (79.4%) adhered to (reported taking) prescribed medications for hypertension, high cholesterol, and/or diabetes (Table 1).

After allowing for study adjustments to the Demographic Assessment for Health Literacy (DAHL) [26], the mean health literacy (DAHL) score amongst respondents was 68.4 (standard deviation (SD) = 14.4), indicating marginal to adequate health literacy. The mean household income to poverty ratio was 2.0 (SD = 1.2), indicating a family income at 200% of the poverty level [27]. The mean prescription medication count was 5.2 (SD = 3.1), with a minimum of one and a maximum of 22 medications.

**Table 1:** Characteristics of the study sample (N = 5513).

Variables (N)	Level	n (%)
Age Group (N = 5513)	65–69 years	1546 (28.0)
	70–74 years	1421 (25.8)
	75+ years	2546 (46.2)
Gender (N = 5513)	Female	2849 (51.7)
	Male	2664 (48.3)
Race ^a^ (N = 5513)	Other	1593 (28.9)
	Non-Hispanic Black	1128 (20.5)
	Non-Hispanic White	2792 (50.6)
Ethnicity ^b^ (N = 5342)	Hispanic	1037 (19.4)
	Non-Hispanic	4305 (80.6)
Education (N = 5492)	<High School Graduate	1727 (31.4)
	≥High School Graduate, but not College Graduate	2696 (49.1)
	College Graduate	1069 (19.5)
Alcohol Consumption Category ^c^ (N = 3899)	Never Drinks	1564 (40.1)
	Light Drinking	2043 (52.4)
	Moderate Drinking	233 (6.0)
	Heavy Drinking	59 (1.5)
Disability Status (N = 5510)	No Disability	3862 (70.1)
	Has Disability	1648 (29.9)
Employment Status ^d^ (N = 5508)	Not Employed	4717 (85.6)
	Employed	791 (14.4)
Household Balanced Meals (N = 5360)	Could Not Afford	790 (14.7)
	Could Afford	4570 (85.3)
Insurance ^e^ (N = 5502)	Medicaid	669 (12.2)
	Medicare	4134 (75.1)
	Other	543 (9.9)
	None	156 (2.8)
Interview Language (N = 5513)	English	4941 (89.6)
	Spanish	572 (10.4)
Lower Social Class ^f^ (N = 4947)	Not Lower Social Class	2945 (59.5)
	Lower Social Class	2002 (40.5)
Marital Status (N = 5509)	Not Married	2494 (45.3)
	Married	3015 (54.7)
Smoking Status (N = 2788)	Does Not Smoke	2279 (81.7)
	Smokes	509 (18.3)
Usual Place for Healthcare (N = 5513)	Does Not Have Usual Place	142 (2.6)
	Has Usual Place	5371 (97.4)
Usual Place for Healthcare Type (N = 5365)	Clinic or Health Center	1101 (20.5)
	Doctor’s Office or HMO	3940 (73.4)
	Hospital Emergency Room	105 (2.0)
	Hospital Outpatient	155 (2.9)
	Other	64 (1.2)
Told By Doctor to Take Prescription for High Blood Pressure (N = 4401)	No	61 (1.4)
	Yes	4340 (98.6)
Told By Doctor to Take a Prescription for High Cholesterol (N = 4814)	No	1198 (24.9)
	Yes	3616 (74.6)
Doctor Told You Have Diabetes (N = 5509)	No	3700 (67.2)
	Yes	1809 (32.8)
Overall Adherence (N = 5513)	Not Adherent	1136 (20.6)
	Adherent	4377 (79.4)

Abbreviations: HMO—health maintenance organization. ^a^ The “Other” race category contains those respondents who did not identify as Non-Hispanic Black or Non-Hispanic White. The “Other” race category included Mexican American [541 (9.8%)], Other Hispanic [496 (9.0%)], Non-Hispanic Asian [385 (7.0%)], and Other Races—including Multiracial [171 (3.1%)]. ^b^ Ethnicity categories were developed from NHANES race/Hispanic origin categories. The “Hispanic” group included respondents identified as Mexican American or other Hispanic. The “non-Hispanic” group included respondents who were classified as Non-Hispanic White, Non-Hispanic Black, or Non-Hispanic Asian. Respondents identified as Other Race—including Multiracial—were categorized as missing. ^c^ Alcohol consumption categories were calculated using responses for the number of days alcoholic drinks were consumed annually, the number of drinks consumed on those drinking days, and guidelines from previous literature [28]. ^d^ Not employed included those reporting that they were not working at a job or business, looking for work, or retired. Those who reported working at a job or business were employed. ^e^ Respondents with Medicaid as at least one source of health insurance were included in the “Medicaid” category, respondents with Medicare (but not Medicaid) as at least one source of insurance were included in the “Medicare” category, and all other respondents without Medicaid or Medicare were included in the “Other” category. The insurance types included in the “Other” category included private insurance, Medi-Gap, military healthcare, state-sponsored health plans, other government insurance, and single-service health plans. ^f^ Respondents with annual family incomes of USD 25,000 or less were classified as lower social class.

As shown in Table 2, bivariate analyses of categorical predictors revealed significant differences in adherence to medications based on structural determinants, including ethnicity (*p* = 0.038), gender (*p* = 0.009), and lower social class status (*p* = 0.023), as well as intermediary determinants, including the level of alcohol consumption [28] (*p* = 0.004), disability status (*p* = 0.014), ability to afford balanced meals for the household (*p* < 0.001), insurance (*p* = 0.010), marital status (*p* = 0.020), and whether or not they had a usual place for healthcare (*p* < 0.001). None of the continuous variables in this initial analysis were significant.

Lower social class, household balanced meals, ethnicity, and education predictors were excluded from the multivariable analysis due to multicollinearity, while gender and marital status were combined due to significant interactions. This analysis (Table 3) revealed that overall significant differences in medication adherence existed based on two intermediary determinants: alcohol consumption and usual place for healthcare. Alcohol consumption was significantly associated with overall medication adherence (*p* = 0.034), with an increasing trend in odds of medication adherence as consumption increases. The odds of being adherent to prescribed medications were approximately 330% higher for those individuals who usually went to a doctor’s office or health maintenance organization (HMO) for healthcare when compared to those who do not have a usual place to go for healthcare (*p* < 0.001) and almost 280% higher for those individuals who usually went to a clinic/health center for healthcare when compared to those who did not have a usual place to go (*p* ≤ 0.001).

## 4. Discussion

This study examined the association between different domains of SDoH and medication adherence among older adults with concomitant diagnoses of high blood pressure, high cholesterol, and/or diabetes in the US using an integrated SDoH framework [23]. It was hypothesized that structural and intermediate determinants of health would be associated with medication adherence. Study inferences summarize the influence of these upstream factors on older adults’ medication adherence, with valuable implications for public health, policy, pharmacy practice, and future research.

The study revealed that ethnicity and several indicators of lower socioeconomic status, including insurance, social class, and ability to afford balanced meals, were significantly associated with medication adherence. Previous studies have also established that medication adherence is lower among racial/ethnic minorities, individuals with no insurance, and people in lower socioeconomic classes [29,30]. These factors were mostly attributed to cost-related medication nonadherence, which is highly pronounced in older adults and racial/ethnic minority groups. Almost 1 in 5 older adults reported cost-related medication non-adherence in 2022, which can subsequently result in increased healthcare utilization and poor clinical outcomes [31,32]. Economic downturns can constrain the ability to meet basic human needs such as food, housing, clothing, and transportation, and patients may resort to foregoing medication acquisition to meet their needs [33,34,35,36]. Hence, pharmacists and other health professionals are encouraged to screen for the SDoH factors associated with medication adherence during clinical assessment using validated SDoH screening instruments while adopting safety net referral programs to achieve longstanding medication adherence outcomes [33].

Multivariable analysis partially supported the hypothesis, as only intermediary determinants of health remained significantly associated with medication adherence. A plausible rationale is that each variable was calculated as if the remaining predictors were held constant and reported independently from associations with other determinants [18]. Alcohol consumption had a significant positive association with medication adherence in this study, which is contrary to most evidence that alcohol consumption has a negative association with adherence [37,38,39,40]. A plausible reason for such a positive correlation may be due to self-report bias from NHANES respondents during data collection. There may be several instances of overreporting or underreporting of drinking status culminating in a skewed output. However, recent qualitative studies revealed that study participants consume considerable amounts of alcohol while taking long-term medications [41,42]. This discordant evidence opens a gap for researchers to leverage a mixed-method approach to determine the interaction between self-reported alcohol usage and medication adherence. This is to determine the drinking behavior of patients with comorbidities who are taking many medications. Pharmacists should inquire about the alcohol-drinking behavior of patients during assessment and consciously educate patients on its interactions with several medications after disclosure. A typical screening tool used in clinical practice is the US Alcohol Use Disorders Identification Test—Consumption (USAUDIT-C), which identifies patients who are heavy drinkers [43]. If addiction is present, then patients should be advised to seek behavioral therapy.

The likelihood of positive medication adherence was higher among study participants who visited clinics, HMOs, or doctors’ offices to access care. Such patients frequently contact pharmacists and other healthcare professionals who design therapeutic plans aimed at preventing medication nonadherence. This can be a substantial SDoH factor to consider when designing and evaluating interventions for populations living in medically underserved areas, which are characterized by few primary care providers [44]. It has been shown that patients living in such areas have a higher rate of abandoning quality-measured prescriptions compared to those living in areas not considered medically underserved, which is indicative of prescription access disparity [45]. It is pertinent to implement strategies that promote pharmacists’ patient care processes, such as medication therapy management, appointment-based models, and pharmacist collaborative practice agreements, to sustain visit regularization and improve medication adherence in these areas [46]. Also, the bipartisan Pharmacy and Medically Underserved Areas Enhancement Act should be adopted to allow pharmacists to be reimbursed for certain healthcare services under Medicare Part B in medically underserved areas with positive implications for healthcare access [47]. The act would specifically grant pharmacists provider status for Medicare patients, which will ensure that they are optimally reimbursed for their services. Via this act, licensed and practicing pharmacists would be reimbursed at 85% of the rate reimbursed to physicians under Medicare Part B in their state if they render their services in a medically underserved area. The implication is that beneficiaries will have greater access to pharmaceutical care when physicians are in short supply [48]. The adoption of such a bill will help promote sustainable medication adherence programs among health disparity populations and extend the reach of pharmacists in actively providing comprehensive pharmaceutical care.

This study is not without limitations. Due to limited resources, this initial study is limited to the SDoH factors specified in the conceptual framework and captured in the NHANES database; however, a typical variable such as medication cost is not included as a barrier to medication access during analyses because it is not available in the NHANES database. The adherence measure used in the analysis was self-reported, which was not in alignment with standardized metrics used in pharmacy practice. Given that the datasets utilized in this study were collected prior to the COVID-19 pandemic, it is possible that healthcare access and medication adherence were relatively stable compared to the pandemic era. Consequently, interpretation of the findings may be constrained, as the pandemic disrupted access to health and social services. Furthermore, the pandemic triggered economic downturns that had direct or indirect impacts on several social determinants of health (SDoH), thereby limiting the generalizability of the results obtained from this study. Future research should utilize other databases that include these data, such as the Medical Expenditure Panel Survey (MEPS). These study findings are relevant to older adults with any combination of hypertension, cholesterol, and diabetes. There could be differences in SDoH associated with medication adherence by specific disease states and age groups, warranting more research.

## 5. Conclusions

Study findings highlight public health, policy, pharmacy practice implications, and opportunities for interventions on the prioritized SDoH most likely to impact medication adherence among older US adults. Bivariate analyses provide strong evidence that structural and intermediate determinants of health are associated with medication adherence. Multivariable analysis partially supports the hypothesis. The odds of older adults being adherent to prescribed medications for hypertension, high cholesterol, and/or diabetes are higher among individuals who have a usual place for healthcare. Legislative measures, such as the Pharmacy and Medically Underserved Areas Enhancement Act, could improve healthcare access and medication adherence in medically underserved areas. Additionally, recent pharmacy SDOH intervention measures include integrating community health workers or cross-training pharmacy technicians as community health workers who serve as trusted liaisons between health and social resources while having a close understanding of the communities they serve. Future research should further investigate the reasoning for the observed increasing medication adherence trend with increased alcohol consumption and build on the current study by examining potential nuances in SDoH associations with medication adherence by disease state and age group.

## Figures and Tables

**Table 2:** Bivariate analyses of overall high blood pressure, high cholesterol, and/or diabetes medication adherence with categorical predictors (N = 5513).

				Medication Adherence		
				Adherent	Not Adherent	
				N = 4377	N = 1136	
Determinant Type	Determinant	Study Variable	Level	N (%)	N (%)	*p*-Value
Structural Determinants	Gender	Gender *	Female	2243 (78.7)	606 (21.3)	0.009 ^a^
			Male	2134 (80.1)	530 (19.9)	
	Race/Ethnicity	Race *	Black	877 (77.7)	251 (22.3)	0.194
			Other	1259 (79.0)	334 (21.0)	
			White	2241 (80.3)	551 (19.7)	
	Race/Ethnicity	Ethnicity *	Hispanic	798 (77.0)	239 (23.0)	0.038 ^a^
			Not Hispanic	3440 (79.9)	865 (20.1)	
	Education	Education *	<HS Grad	1375 (79.6)	352 (20.4)	0.124
			College Grad	865 (80.9)	204 (19.1)	
			HS Grad	2121 (78.7)	575 (21.3)	
	Occupation	Employment Status	Not Employed	3740 (79.3)	977 (20.7)	0.357
			Employed	633 (80.0)	158 (20.0)	
	Social Class	Lower Social Class *	Not Lower Social Class	2372 (80.5)	573 (19.5)	0.023 ^a^
			Lower Social Class	1561 (78.0)	441 (22.0)	
Intermediary Determinants	Biological Factor	Age Group	65–69	1215 (78.6)	331 (21.4)	0.343
			70–74	1157 (81.4)	264 (18.6)	
			75+	2005 (78.8)	541 (21.2)	
	Material Circumstance	Household	Could Afford	3684 (80.6)	886 (19.4)	<0.001 ^a^
		Balanced Meals *	Could Not Afford	574 (72.7)	216 (27.3)	
	Psychosocial	Marital Status *	Not Married	1926 (77.2)	568 (22.8)	0.020 ^a^
			Married	2447 (81.2)	568 (18.8)	
	Behaviors	Smoking Status	Does Not Smoke	1807 (79.3)	472 (20.7)	0.428
			Smokes	401 (78.8)	108 (21.2)	
	Behaviors	Alcohol	Heavy	51 (86.4)	8 (13.6)	0.004 ^a^
		Consumption Category *				
			Light	1644 (80.5)	399 (19.5)	
			Moderate	196 (84.1)	37 (15.9)	
			Never	1209 (77.3)	355 (22.7)	
	Medication Access— Provider Availability	Usual Place for Healthcare *	Does Not Have Usual Place	82 (57.7)	60 (42.3)	<0.001 ^a^
			Has Usual Place	4295 (80.0)	1076 (20.0)	
	Medication Access—Disability Status	Disability Status *	No Disability	3140 (81.3)	722 (18.7)	0.014 ^a^
			Has Disability	1234 (74.9)	414 (25.1)	
	Medication Access—Provider Availability	Usual Place for Healthcare Type *	Clinic/Health Center	865 (78.6)	236 (21.4)	0.091
			Doctor Office	3190 (81.0)	750 (19.0)	
			Hospital ER	73 (69.5)	32 (30.5)	
			Hospital OP	116 (74.8)	39 (25.2)	
			Other	47 (73.4)	17 (26.6)	
	Medication Access—Insurance	Insurance *	Medicaid	511 (76.4)	158 (23.6)	0.010 ^a^
			Medicare	3285 (79.5)	849 (20.5)	
			None	122 (78.2)	34 (21.8)	
			Other	450 (82.9)	93 (17.1)	
	Medication Access—Language	Interview Language	English	3922 (79.4)	1019 (20.6)	0.962
			Spanish	455 (79.5)	117 (20.5)	

Abbreviations: HS—high school; Grad—graduate; ER—emergency room; OP—outpatient. ^a^ Predictors were significant at the alpha = 0.05 level. * Predictors with *p*-values less than 0.20 were considered in the multivariable analysis.

**Table 3:** Multivariable analysis of overall high blood pressure, high cholesterol, and/or diabetes medication adherence (N = 3887).

Variables (N = 3887)	Odds Ratio (95% CI)	*p*-Value
Alcohol Consumption Category		**0.034** ^b^
Alcohol Consumption Category (Light vs. Never)	1.164 (0.881, 1.538)	0.281
Alcohol Consumption Category (Moderate vs. Never)	1.657 (1.085, 2.531)	**0.020** ^b^
Alcohol Consumption Category (Heavy vs. Never)	2.866 (1.122, 7.318)	**0.028** ^b^
Disability Status (Disability vs. No Disability)	0.884 (0.659, 1.185)	0.404
Insurance		0.080
Insurance (Medicaid vs. None)	0.885 (0.423, 1.853)	0.744
Insurance (Medicare vs. None)	0.926 (0.502, 1.709)	0.804
Insurance (Other vs. None)	1.597 (0.791, 3.224)	0.189
Gender/Marital Status ^a^		0.097
Gender, Marital Status (Female Married vs. Female Not Married)	1.257 (0.855, 1.847)	0.241
Gender, Marital Status (Male Married vs. Female Not Married)	1.337 (0.993, 1.801)	0.055
Gender, Marital Status (Male Not Married vs. Female Not Married)	1.492 (1.050, 2.118)	**0.026** ^b^
Race		0.566
Race (Black vs. White)	0.902 (0.727, 1.121)	0.349
Race (Other vs. White)	0.908 (0.694, 1.187)	0.475
Usual Place for Healthcare ^c^		**0.001** ^b^
Usual Place for Healthcare (Clinic/Health Center vs. None)	3.796 (1.904, 7.569)	**<0.001** ^b^
Usual Place for Healthcare (Doctor Office or HMO vs. None)	4.297 (2.274, 8.118)	**<0.001** ^b^
Usual Place for Healthcare (Emergency Room vs. None)	2.341 (0.937, 5.850)	0.068
Usual Place for Healthcare (Hospital Outpatient vs. None)	4.068 (1.674, 9.888)	**0.002** ^b^
Usual Place for Healthcare (Other vs. None)	1.964 (0.593, 6.510)	0.265

Abbreviations: HMO—health maintenance organization; CI—confidence interval. ^a^ Predictors with multicollinearity were combined into a single predictor variable. ^b^ Predictors were significant at the alpha = 0.05 level. ^c^ Whether the respondent had any usual place for healthcare and the specific type of usual place for healthcare were combined into one predictor variable as all responses for a usual place for healthcare type had a response of “Yes” for a usual place for healthcare. The combined variable includes original responses for the usual healthcare place type variable plus the “Does Not Have Usual Place” level from the usual healthcare binary variable.

## Data Availability

All NHANES datasets supporting the conclusions of this article, interpretations, computations, and mapping of study variables to conceptual framework elements are available in the Figshare data dictionary: https://doi.org/10.6084/m9.figshare.21947018 (accessed on 2 February 2025).
